# Immunohistochemical Studies of Cytokeratins and Differentiation Markers in Bovine Ocular Squamous Cell Carcinoma

**DOI:** 10.3390/vetsci7020070

**Published:** 2020-05-29

**Authors:** Helena Vala, Tânia Carvalho, Carlos Pinto, Maria A. Pereira, João R. Mesquita, Maria C. Peleteiro, Lluís Ferrer, Dolores Fondevila

**Affiliations:** 1 Agrarian School of the Polytechnic Institute of Viseu, Quinta da Alagoa-Estrada de Nelas Ranhados, 3500-606 Viseu, Portugal; hvala@esav.ipv.pt; 2 Centre for the Research and Technology of Agro-Environmental and Biological Sciences (CITAB), University of Trás-os-Montes and Alto Douro, 5001-801 Vila Real, Portugal; 3 Instituto de Medicina Molecular, Faculdade de Medicina da Universidade de Lisboa, Av. Professor Egas Moniz, 1649-028 Lisboa, Portugal; taniacarvalho@medicina.ulisboa.pt; 4 Faculdade de Ciências Agrárias e do Ambiente—Universidade dos Açores, Rua Capitão João d’Ávila—Pico da Urze, 9700-042 Angra do Heroísmo, Portugal; carlos.a.pinto@uac.pt; 5 Global Health and Tropical Medicine (GHTM), Instituto de Higiene e Medicina Tropical (IHMT), Universidade Nova de Lisboa (UNL), R. da Junqueira 100, 1349-008 Lisboa, Portugal; 6 Instituto de Ciências Biomédicas Abel Salazar, Universidade do Porto, Rua de Jorge Viterbo Ferreira 228, 4050-313 Porto, Portugal; jrmesquita@icbas.up.pt; 7 Faculdade de Medicina Veterinária, Universidade de Lisboa, Avenida da Universidade Técnica, 1300-477 Lisboa, Portugal; mcpelet@fmv.ulisboa.pt; 8 Departament de Medicina i Cirurgia Animals, Facultat de Veterinària, Universitat Autònoma de Barcelona, 08193 Barcelona, Spain; Lluis.Ferrer@uab.cat (L.F.); dolors.fondevila@uab.cat (D.F.)

**Keywords:** bovine, cytokeratin, immunohistochemistry, involucrin, ocular squamous cell carcinoma, profilaggrin

## Abstract

Bovine Ocular Squamous Cell Carcinoma is considered the most common bovine tumour, causing significant economic losses, mainly by abattoir condemnations. To obtain a better insight into the genesis and neoplastic transformation, 19 samples collected at slaughter from Holstein Friesian cattle and diagnosed as Ocular Squamous Cell Carcinoma were studied. Tumours were histologically classified into three categories: poorly (26.3%), moderately (26.3%), and well differentiated (47.4%). Expression of keratins (MNF116 and LP34) and of cornified envelope precursors (involucrin and profilaggrin) was studied. Expression of MNF116 was observed in all carcinomas. LP34 immunostaining was seen in all but three carcinomas, one from each degree. Involucrin immunoreaction was observed in all but one poorly differentiated carcinoma. Profilaggrin was present in only two moderately differentiated carcinomas, in all but one well differentiated, and in all but one poorly differentiated. MNF116 is a useful marker to confirm the epithelial origin of the tumour and stain most neoplastic cells in these tumours. The expression of involucrin and LP34 demonstrates that, in all tumours, cells have reached the final program of differentiation, regardless of the grade. The expression of profilaggrin could indicate molecular changes during malignant transformation but their expression does not seem to be of diagnostic value.

## 1. Introduction

Ocular Squamous Cell Carcinoma (OSCC) is a general designation for a group of primary neoplasms of keratinocytes emerging from ocular tissues, especially the eyelids and particularly the third eyelid [1]. Bovine Ocular Squamous Cell Carcinoma (BOSCC) has been diagnosed all over the world with high incidence, and it is considered the most common bovine tumour and the one which causes the most significant economic losses, mainly by abattoir condemnations [1,2,3,4,5,6,7,8]. This tumour is described mostly in aged animals from Hereford and Holstein or derived breeds, among others [1,2,4,5,8]. In Portugal, the frequency of this tumour is particularly high in the Azores. On S. Miguel Island, large numbers of cattle affected with OSCC are rejected for consumption at slaughter and OSCC is the second most frequent neoplasia, after urinary bladder tumours, representing 21% of all cases of rejection due to neoplasia [9,10,11].

BOSCC arises from plaque and papilloma or may develop spontaneously without progression from benign lesions. BOSCC is an invasive and chronically progressing tumour that may spread to the parotid lymph node and invade the bone of the orbit and adjacent structures [2,3]. Histologically, OSCC may range from well to poorly differentiated or anaplastic carcinoma, but like many epithelial tumours, it may have a heterogeneous population and could display different histological features in the same neoplasia. Hence, the study of the antigenic expression in the different patterns is useful to develop a deeper understanding of the cellular behaviour and deregulation [12,13,14].

The expression of distinct intermediary filaments is further associated with distinct steps of malignant transformation [15]. Cytokeratins (CK), cytoskeleton proteins, are expressed exclusively by epithelial cells during differentiation of keratinocytes, and are preserved during malignant transformation, as in squamous cell carcinoma (SCC) [16]. Involucrin and filaggrin are considered markers of keratinocyte terminal differentiation. Synthesis of involucrin starts in *stratum spinosum* keratinocytes, after they have stopped their division, and is considered an early marker of terminal differentiation [14,17,18,19,20,21,22,23]. Involucrin expression could distinguish benign lesions from SCC [24,25]. Profilaggrin is synthesised in the upper *stratum spinosum* or *granulosum*, stored in the keratohyalin granules, and posteriorly broken down into filaggrin [26,27]. Filaggrin and profilaggrin, expressed by keratinocytes of the granular cell layer of normal keratinized epithelia, are considered later differentiation markers [22,28].

To the best of our knowledge, there are several immunohistochemical (IHC) studies focusing on pre-neoplastic lesions and epidermic/oral SCC in man [29,30,31,32] and in small animals [33,34,35] but very few in farm animals [1,36,37]. Since every effort is needed to improve knowledge on the biology of tumours and search for predictive factors on the prognosis, the aim of this study was to evaluate the histological and IHC features of BOSCC. The histological pattern was compared with the expression of keratinocytes differentiation proteins, CK, involucrin, and filaggrin, to facilitate the histopathologic classification and biological behaviour on the routine diagnosis of the BOSCC.

## 2. Materials and Methods

### 2.1. Samples

Nineteen eye tumour samples were collected at slaughter, on São Miguel Island, Azores, from Holstein Friesian cattle. Samples were fixed in 10% neutral-buffered formalin, for a maximum of 48 h, embedded in paraffin wax, and 3 μm sections were stained for routine histopathological diagnosis with haematoxylin and eosin (H/E).

### 2.2. Histopathology

Before histological processing, all tumours were evaluated and classified according to macroscopic features based on World Health Organization (WHO) classification [38]. All samples were examined by light microscopy and classified according to morphological criteria [11,38,39,40]. These criteria included: (1) Cellular phenotype evaluating the proportion between basal-like keratinocytes and well-differentiated keratinocytes, organisation pattern in islands, trabeculae, or cords, showing central squamous differentiation, degree of keratinisation, namely evaluating the presence, number, and dimension of keratin pearls, and presence of pleomorphic and atypical cells and presence of dysqueratotic and acantolytic cells. (2) Mitotic index refereed as the number of mitotic figures per 10 high-power fields, ranging from 0–2 (Low), 3–6 (Medium), and >6 (High). (3) Invasiveness, evaluating infiltration of the surrounding tissues. According to their degree of differentiation, mitotic index, and infiltration of the surrounding tissues, the BOSCC were classified as described elsewhere into well differentiated (WD), moderately differentiated (MD), and poorly differentiated (PD) (Table 1). 

### 2.3. Immunohistochemistry (IHC)

IHC staining was performed using the streptavidin-biotin-peroxidase method. 

Deparaffinized and rehydrated 3 μm thick tissue sections were mounted on Vectabond (VECTOR, Burlingame, CA, USA)-coated glass slides. Endogenous peroxidase activity was abolished with 0.5% hydrogen peroxide in methanol for 30 min and non-specific staining was blocked by pre-incubation with normal swine serum (1:5 dilutions, 60 min).

To evaluate keratin expression, two monoclonal antibodies (DAKO, Glostrup, Denmark) were used: MNF116 and LP34. The distribution of cornified envelope precursors was studied using two antibodies: monoclonal antibody anti-human involucrin (NOVOCASTRA, Newcastle upon Tyne, UK) and polyclonal antibody anti-human profilaggrin (ZYMED, South San Francisco, CA, USA). Sections were washed in Tris-buffered saline (TBS, pH 7.4) and incubated with Multilink (DAKO), diluted 1:100, for 60 min at room temperature, as secondary antibodies. After incubation with streptavidine-biotinylated horseradish peroxidase complex (DAKO) for 60 min, reaction was developed with 0.075% 3.3 diaminobenzidine tetrahydrochloride (SIGMA) and 0.02% hydrogen peroxide for 10 min (Table 2). Finally, sections were counterstained with Mayer’s hemalumen (MERCK, Darmstadt, Germany), dehydrated, and mounted in Entelan (MERCK). To assess the number of positively labelled cells, a semi-quantitative scoring system was used for each antibody: (−) absence of labelling, (+) <25% of cells labelled, (++) 25%–50% of cells labelled, (+++) 50%–75% of cells labelled, and (++++) >75% of cells labelled.

Bovine and human normal skin was used as positive control, and for negative controls, the primary antibodies were replaced by phosphate-buffered saline. 

### 2.4. Statistical Analysis

In order to verify a potential difference in the expression of the different markers (MNF, LP34, profilaggrin and involucrin) between the three histological differentiation groups of BOSCC (WD, MD, and PD), data obtained from the expression of the different markers were submitted to analysis of variance (ANOVA). Pearson’s correlation test was used to determine if any correlation was present between the expressions of the markers. For the statistical analysis, the software SPSS 25^®^ (IBM corp., New York, NY, USA) was used. Differences were considered statistically significant when the computed *p*-value was less than 0.05.

### 2.5. Ethics

Ethical procedures were approved by Portuguese Foundation for Science and Technology (FCT) (PRAXIS/BD/18347/98) and by the Interdisciplinary Centre for Research in Animal Health (CIISA), Faculty of Veterinary Medicine, University of Lisbon.

## 3. Results

### 3.1. Clinical Data 

All tumours collected were from Holstein Friesian breed with ages ranging from 4 to 10 years (mean 7.5 years). Eight tumours had origin on the eyelids, eight on the third eyelid, and three involved the whole ocular region (Table 3).

### 3.2. Histopathological Diagnosis

**Figure 1:**
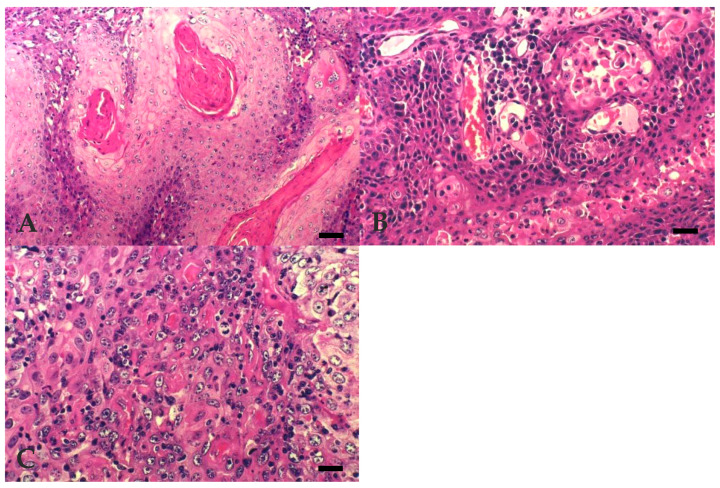
Bovine Ocular Squamous Cell Carcinoma. (**A**) WD: extensive keratin pearl formation. Bar, 50 µm. (**B**) MD: acantolytic cysts. Bar, 25 µm. (**C**) PD: individual cell keratinisation with more pleomorphic and atypical cells. Bar, 25 µm. Hematoxylin Eosin (HE).

The distribution of BOSCC regarding the degree of differentiation and invasiveness are summarised in Table 3. All 19 tumours sampled were diagnosed as OSCC. WD BOSCC 9/19 (47.4%) (Figure 1A) were characterised by the presence of numerous large keratin pearls and large islands, trabeculae, or cords formation, with central squamous differentiation. These tumours showed small basal-like cells at the periphery and well-differentiated keratinocytes, with homogenous eosinophilic cytoplasm and clear intercellular bridges in the central areas. MD BOSCC 5/19 (26.3%) (Figure 1B) were characterised by the presence of a moderate degree of keratinisation and differentiation, small to medium sized keratin pearls, acantolytic cysts, smaller islands, and squamous differentiation, but with an increased number of poorly differentiated cells. PD BOSCC 5/19 (26.3%) (Figure 1C) showed individual cell keratinisation, few small sized islands, and poor cellular differentiation, with predominance of areas with more pleomorphic and atypical cells. 

Dysqueratotic and acantolytic cells were present in the three degrees of differentiation. Regarding invasiveness, the highest grade corresponded to invasion of the deepest skin layers, far from the main tumour, the intermediate grade to neoplasms with small independent islands of neoplastic tissue surrounding the main tumour, and the lowest grade was assigned to neoplasms with minimal signs of invasion of the surrounding tissues. Mitotic index was low in 7 WD, 4 MD, and 2 PD, medium in 1 WD and in 2 PD, and high in 1 WD, 1 MD, and 1 PD (Table 3). 

### 3.3. Immunohistochemistry

The results of immunohistochemistry obtained with all antibodies in BOSCC are summarized in Table 3. 

**Figure 2:**
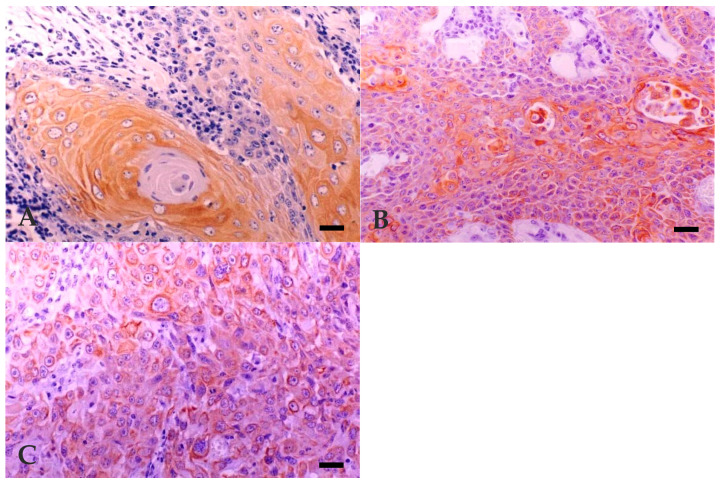
Expression of cytokeratin marker MNF116 in BOSCC. (**A**) WD: cytoplasmic diffuse and intense reaction—more intense in well-differentiated keratinocytes. Bar, 25 µm. (**B**) MD: cytoplasmic diffuse and intense reaction—more intense in well-differentiated keratinocytes. Bar, 25 µm. (**C**) PD: cytoplasmic diffuse and intense reaction in most neoplastic cells, including those of pleomorphic areas. Bar, 25 µm. Immunohistochemistry using avidin-biotin complex method, Mayer’s Hematoxylin counterstain.

In bovine and human normal skin, MNF116 intensively stained the *stratum basale*, mildly stained the lower *stratum spinosum*, was absent from upper *stratum spinosum* and *stratum granulosum*, and intensively stained the simple epithelium of the cutaneous glands. MNF116 immunoreactivity was observed in all neoplasias (100%), irrespective of their degree of differentiation (Figure 2A–C). Intense diffuse cytoplasmatic reaction was present in most of the cells, including the pleomorphic cells of poorly differentiated BOSCC. Reaction was more intense in well-differentiated keratinocytes or squamous-like phenotype cells, in the small basal-like cells, located at the periphery of the tumour trabeculae or cords, and in the dysqueratotic and acantolytic cells. 

**Figure 3:**
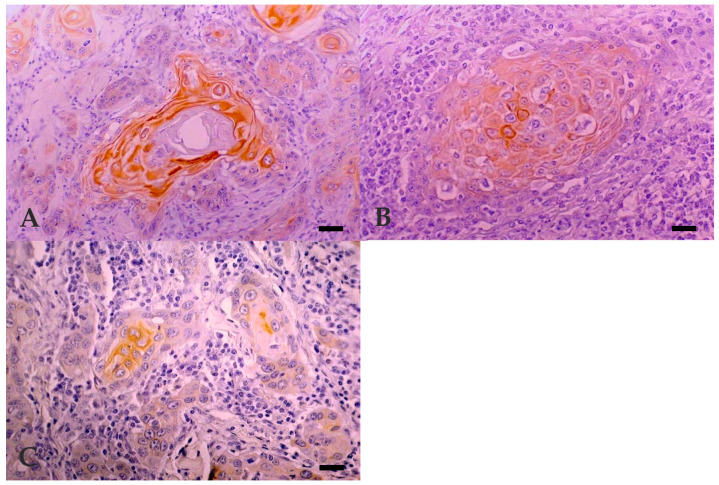
Expression of cytokeratin marker LP34 in BOSCC. (**A**) WD: cytoplasmic diffuse and intense immunoreactivity, seen mostly in well-differentiated tumour cells but also in dysqueratotic and acantolytic cells. Bar, 50 µm. (**B**) MD: cytoplasmic diffuse and reaction in fewer well-differentiated keratinocytes. Bar, 25 µm. (**C**) PD: cytoplasmic diffuse and intense reaction, only in a limited number of well-differentiated tumour cells in the centre of islands. Bar, 25 µm. Immunohistochemistry using avidin-biotin complex method, Mayer’s Hematoxylin counterstain.

In bovine and human normal skin, LP34 intensively stained the suprabasal layers and was absent from *stratum basale*. LP34 immunoreactivity was observed in all BOSCC except 3, 16/19 (84.2%), 1 in each of the three groups of differentiation. Reactivity was cytoplasmatic diffuse and intense, especially in well-differentiated cells with squamous phenotype, whereas basal-like cells were negative (Figure 3A–C). Dysqueratotic and acantolytic cells were also positive in all degrees of differentiation. Intensity and percentage of labelled cells diminished as the degree of differentiation of the BOSCC decreased. However, the expression of LP34 between the three histological differentiation groups was not statistically different (*p* = 0.365). Most of the BOSCC studied revealed co-expression of simple (LP34) and stratified (MNF116) cytokeratins markers.

**Figure 4:**
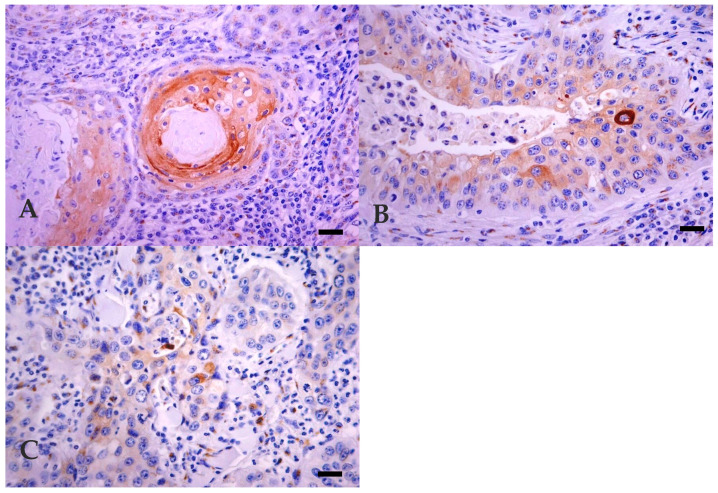
Expression of involucrin in BOSCC. (**A**) WD: cytoplasmic diffuse and intense immunoreactivity, seen mostly in well-differentiated tumour cells. Bar, 25 µm. (**B**) MD: cytoplasmic diffuse and intense reaction, only in a limited number of well-differentiated tumour cells but also in dysqueratotic and acantolytic cells. Bar, 25 µm. (**C**) PD: cytoplasmic diffuse and intense reaction, only in a limited number of well-differentiated tumour cells. Bar, 25 µm. Immunohistochemistry using avidin-biotin complex method, Mayer’s Hematoxylin counterstain.

In bovine and human normal skin, an intense immunoreaction with involucrin was seen in the upper *stratum spinosum* and *stratum granulosum* and was observed in all but one PD BOSCC 18/19 (94.7%). Cytoplasmic diffuse and intense reaction was observed only in a limited number of well-differentiated tumour cells, independently of the degree of differentiation, coincident with squamous cells surrounding keratin pearls. Reaction was observed in dysqueratotic cells in two BOSCC, and acantolytic cells in another two BOSCC (Figure 4A–C). 

**Figure 5:**
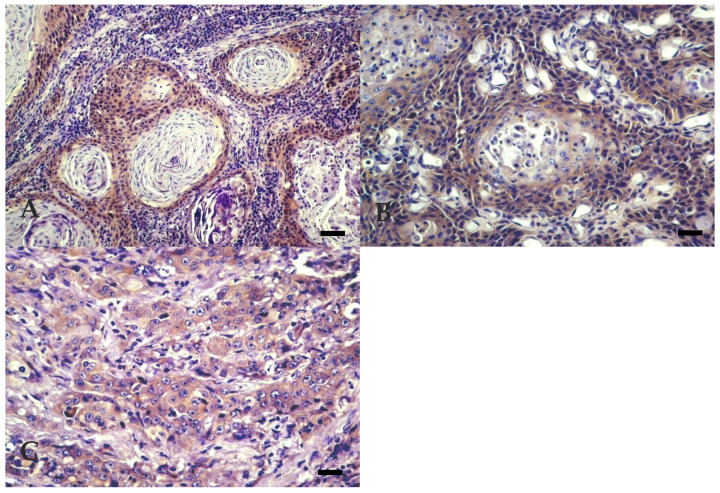
Expression of profilaggrin in BOSCC. (**A**) WD: granular cytoplasmatic and intense reaction, observed in all cellular types, including basal-like cells. Bar, 50 µm. (**B**) MD: granular cytoplasmatic and intense reaction in all cellular types, including basal-like cells. Bar, 25 µm. (**C**) PD: unspecific granular cytoplasmatic reaction observed in all cellular types, including basal-like cells. Bar, 25 µm. Immunohistochemistry using avidin-biotin complex method, Mayer’s Hematoxylin counterstain.

In bovine and human normal skin, profilaggrin showed a granular cytoplasmatic reaction only in *stratum granulosum*. In BOSCC, profilaggrin expression was exclusively seen in two MD BOSCC, all but one WD BOSCC, and all but one PD BOSCC 14/19 (73.7%). In all of these, a granular cytoplasmatic reaction was observed in all cellular types, including basal-like cells (Figure 5A–C). However, in 7 BOSCC, the reaction was irregular and heterogeneous, with some negative areas alternating with focally intensely stained areas. The number of labelled cells was also variable. Immunoreaction was never observed in dysqueratotic or acantolytic cells in any tumours. The expression of profilaggrin between the three histological differentiation groups of BOSCC was not statistically different (*p* = 0.268). Pearson’s correlation test did not reveal any correlation between expression of LP34 and profilaggrin (r = 0.06).

## 4. Discussion

The predominant ocular localisation of the BOSCC studied was eyelids, and especially the third eyelid. The origin of the three tumours involving the whole ocular region was unknown. The cattle breeding system with all-year-round exposure to ultraviolet radiation determines an increased susceptibility to the carcinogenic effect of sunlight in the Azores, as reported by other authors in other parts of the globe [1,8,41]. Since these animals stay in pasture all year around, with prolonged exposition to daylight, and because the effect of ultraviolet radiation is cumulative, tumours were mainly observed in adult or old cattle [8,42].

The histopathological pattern of the BOSCC is in agreement with that described by other authors [11,38,39,40,43,44,45]. With the exception of some reports which described high mitotic index [45], most referred to it as moderate to high [38,39,43,44]. In most of BOSCC studied here, the mitotic index was low.

The results obtained for MNF116 revealed that this antibody is a useful marker to confirm the epithelial origin of less-differentiated carcinomas. On the other hand, the positive immunostaining of the majority of the neoplastic cells, independently of the cellular type, identified in all three groups of BOSCC, differs from the results obtained in normal skin in which positivity is only seen in keratinocytes from the basal layer. This is in agreement with the fact that keratins characteristic of simple epithelium (like the pair K8/K18), originally restricted to the basal layer of the normal epidermis, are mostly expressed by neoplastic cells in the human SCC [16,22,46,47,48]. On the other hand, keratins recognised by this antibody (like K6 and K17) are considered markers of hyperproliferative keratinocytes [16,48,49,50], present in hyperplastic and neoplastic lesions of the squamous epithelium.

Only MNF116 stained small basal-like cells, which are cells with high turnover that resemble those of the basal layer of epidermis, as reported by some authors [46,47,48]. The results obtained with MNF116 are also consistent with those of other authors, which propose that the malignancy grade and aggressive biological behaviour of SCC reflect a reduction of high-molecular-weight keratins’ expression, typical of more differentiated keratinocytes, and an increase of the low-molecular-weight keratins, specific for simple epithelia and recognised by this antibody [15,31,48]. So, in SCC, the expression of simple epithelium keratins, by most neoplastic cells, including those with a more differentiated phenotype, may translate their ability to invade, their metastatic potential, and could also be associated with poor prognosis [29,48,51,52]. This staining pattern could help distinguish malignant neoplasias from premalignant or benign ones, which reveal a cytokeratin pattern more similar to that of normal epidermis [29,48,51,52].

It was also demonstrated that simple epithelium keratins might be markers not only of simple epithelium but also of primitive keratinocytes, namely embryonic and foetal keratinocytes [31,48]. The positive reaction obtained in most neoplastic cells, including those in anaplastic areas of PD BOSCC, could also be explained by atavism, suggesting that tumour cells that undergo malignant transformation could express markers from embryonic and foetal keratinocytes. 

The only imunohistochemical profile of SCC in domestic animals was performed in sheep, using two monoclonal antibodies, one against K5 and K8, and the other to K8/K18 and K19 [36]. Results obtained by Perez et al. [36] with the first antibody are similar to our results obtained with the MNF116 antibody, with immunopositivity in the majority of neoplastic cells. However, the antibody against K8/K18 and K19 did not react with the neoplastic cells in the referred study [36].

The results obtained with LP34 are in line with studies performed in humans, which report a positive reaction only in squamous cells surrounding the keratin pearls [29,48]. However, some authors advocate that the criteria of using cytokeratin expression to define relationships between cells and histogenesis of the tumours are premature and may be misleading [53].

On the other hand, as may be evaluated from the results of the present work, simple tumour morphology may be insufficient to corroborate the classification of the BOSCC degrees, as absence of staining with this antibody was obtained in a MD tumour, and also in a WD tumour. This BOSCC presented several keratin pearls but with a small number of concentric cell layers around it, which could mean that the cells suffered abrupt keratinisation, without typical squamous differentiation, probably without reaching the capacity to produce and express keratin filaments characteristic of more differentiated keratinocytes, as cited in a previous study [12]. Also, PD BOSCC that were positive showed pleomorphic and atypical squamous cells that were unable to organise into keratin pearls but could express those keratin filaments, characteristics of well-differentiated keratinocytes, as advocated in a previous study in human SCC [48].

Hence, in what concerns keratins, the co-expression of simple- and stratified-type cytokeratins, seen in most BOSCC studied, confirms the presence of phenotypic characteristics of both cell lineages: basal and squamous types, which undergo variable degrees of differentiation, according to previous statements [13]. Also, expression of cytokeratins typical of non-differentiated keratinocytes and simple epithelia is suggestive of a downregulation of stratified-type cytokeratins, proportional to the reduction in the degree of differentiation, as reported for SCC in humans [15,31,48]. 

The presence of involucrin in WD squamous cells of all three groups of BOSCC reveals that the capacity to form concentric keratin pearls does not correlate with the degree of cell differentiation. Involucrin reaction was found to be proportional to the degree of squamous differentiation and not to the degree of keratinisation in a previous study involving benign and malignant lesions (SCC) of human oral cavity [29]. This fact was not established in our study, since we concluded that all groups presented cells reaching an almost complete program of differentiation, which was not expected, especially in the poorly differentiated BOSCC, however is in line with a previous study concerning the SCC of human skin [48].

A study on human oesophageal SCC indicated involucrin as a powerful biological marker of cellular differentiation and also defended correlation between the histological grade and the involucrin expression, as the percentage of cells expressing involucrin was diminishing from the well to the poorly differentiated SCC [30]. This fact was not well established in our study, since almost all tumours, independently of differentiation grade, revealed a constant and low number of positive cells. However, our results are in agreement with the authors that obtained a low number of stained cells in carcinomas, when compared with papilloma or keratoacantomas [24,25,54,55]. 

By labelling squamous-like cells only, even in PD carcinomas, involucrin immunoreaction determines the epithelial origin of the neoplasia, allowing the distinction from other carcinomas, namely basal cell tumours or other proliferative lesions formed by basaloid keratinocytes, which do not express detectable immunoreactivity for involucrin, except in focal areas of eventual squamoid differentiation, according to previous statements [24]. However, the same was not observed with profilaggrin, which, in our study, labelled all epithelial cells, including basal-like ones, in the BOSCC. 

The results obtained for involucrin and keratins, characteristics of terminal differentiated keratinocytes, were similar between WD BOSCC and normal epidermis, with enhanced differentiation toward the centre of the islands. Nevertheless, even PD BOSCC, characteristically with a large number of atypical squamous cells, invariably displayed normal squamous phenotype. Similar results were also obtained in a previous study with human SCC [29]. 

Since profilaggrin also represents a later marker of terminal differentiation [22,28], its presence in cells that normally do not synthesize this protein in BOSCC was totally unpredictable. The positive reaction obtained in 14 BOSCC (74%) is not in accordance with what was found by other authors that defend the lack of profilaggrin and filaggrin immunostaining as being an indicator of malignancy, since they obtained negative reactions in SCC of human skin, contrasting with intense positivity obtained in benign keratoacanthomas [56]. Our result could reflect cellular changes that take place during malignant transformation, an interpretation also advocated by some authors [29]. 

The fact that several carcinomas showed an irregular intensity reaction could probably reflect a disorderly terminal differentiation in these areas and, consequently, this expression could be a sign of malignancy, meaning that cells express filaggrin inconsistently, not giving any predictable diagnostic pattern, which is also verified by other authors in human similar neoplasias [25,29]. In contrast, a more homogeneous, uniform pattern of moderate intensity reaction was described in benign lesions with differentiation markers [25]. 

The number of BOSCC negative for profilaggrin was higher than with all other antibodies, since profilaggrin and filaggrin are markers that are expressed later in the differentiation process, responsible to the keratin aggregation, have a short life span, and cannot be detectable in hyperproliferative lesions, either benign or malignant [29], which may be due to the type of keratinisation in these tumours. 

With the exception of the LP34 and profilaggrin, all antibodies revealed similar distribution (with respect to the number of positive tumours and intensity of reaction) between the three histological differentiation groups of BOSCC. For this reason, expression of LP34 and profilaggrin were submitted to analysis of variance. Statistical analysis showed no differences between the expression of LP34 or profilaggrin and BOSCC histological classification, although a tendency of higher expression towards high-grade BOSCC was noted. Also, no correlation was found between LP34 and profilaggrin, this fact could be due to the expression of each one in different cell populations, as described above. 

Dysqueratotic and acantolytic cells were stained for MNF116, LP34, involucrin, and were negative for profilaggrin. These results are in accordance with Vigneswaran and co-workers [29], who obtained similar results in human oral SCC. 

In summary, all four commercial human antibodies that were tested reacted in bovine tissues, more precisely in the BOSCC. The MNF116, directed against keratins characteristics of simple epithelium, is a useful marker to confirm the epithelial origin of this neoplasias and its malignancy. It was the only antibody which reacted with most cell types, including small basal-like cells. 

The presence of involucrin and keratins, characteristic of well-differentiated keratinocytes, allows the confirmation of the squamous phenotype and, consequently, the determination of an epithelial neoplasia with a squamous origin. Also, with these antibodies, it was possible to verify that, in all degrees of neoplastic differentiation, cells were present that reached the final program of differentiation, independently of the keratinisation degree observed. 

The presence of profilaggrin in basal-like cells could indicate molecular changes during malignant transformation, also reflecting their malignant potential, but the negative staining obtained in tumours of the three degrees of differentiation and the inconsistent reaction pattern indicates that this antibody is not of value in the diagnosis classification of BOSCC.

## 5. Conclusions

In conclusion, the most common immunophenotype found in BOSCC was positivity for MNF116, LP34, involucrin, and profilaggrin. The strong expression obtained with cytokeratin and involucrin revealed that these are the most specific markers for BOSCC, with profilaggrin being the more unspecific one.

## Figures and Tables

**Table 1:** Bovine Ocular Squamous Cell Carcinoma (BOSCC) histopathological classification regarding degree of differentiation and invasiveness [11,38,39,40].

**Degree of Differentiation**			
	**Keratinisation Degree**	**Pattern**	**Cellular Phenotype**
Well Differentiated	Numerous large keratin pearls	Large islands, trabeculae or cords formation, with central squamous differentiation	Small basal-like cells at the periphery and well-differentiated keratinocytes, with homogenous eosinophilic cytoplasm and clear intercellular bridges in the central areas
Moderately Differentiated	Small to medium sized keratin pearls	Smaller islands and squamous differentiation, acantolytic cysts	Increased number of poorly differentiated cells
Poorly Differentiated	Individual cell keratinisation	Few small sized islands, poor cellular differentiation	Predominance of areas with more pleomorphic and atypical cells
**Degree of Invasiveness**			
	**Non-Invasive**	**Moderately Invasive**	**Very Invasive**
	Minimal signs of invasion of the surrounding tissues	Small independent islands of neoplastic tissue surrounding the main tumour	Invasion of the deepest skin layers, far from the main tumour

**Table 2:** Antibodies and techniques.

Antibody	Clone	Pre-Treatment	Dilution	Incubation Time
Cytokeratins 5, 6, 8, 17, and 19	MNF116 (DAKO)	Tripsin to 0.1% 20′, 37 °C	1:50	Overnight Room temperature
Cytokeratins 5, 6, and 18	LP34 (DAKO)		1:100	
Profilaggrin	Policlonal (ZYMED)		1:150	
Involucrin	SY5 (NOVOCASTRA)	10′, 98 °C in 10 mM citrate buffer (pH 6.0)	1:20	

**Table 3:** Clinical data, tumour anatomic location, histological classification, mitotic index, and immunohistochemical results.

Animal *	Age (Year)	Localization of Tumour	Histological Classification **	Invasiveness ^‡^	Mitosis ^§^	Immunohistochemistry ^†^			
						MNF 116	LP 34	Involucrin	Filaggrin
1	6	Third eyelid	WD	MI	0–20	+++	+++	+	+++
2	5	Eyelid	WD	MI	0-20	+++	++	+	++++
3	8	Third eyelid	WD	NI	>60	++++	+	+	++++
4	4	Eyelid	WD	NI	30–60	++++	++	+	++++
5	10	Third eyelid	WD	VI	0–20	++++	++++	+	++
6	10	Whole ocular region	WD	VI	0–20	+++	−	+	−
7	8	Eyelid	WD	VI	0–20	++++	++	+	+
8	8	Eyelid	WD	NI	0–20	++++	+	+	+
9	7	Third eyelid	WD	VI	0–20	++++	+++	+	+++
10	9	Third eyelid	MD	MI	>60	++++	+	+	++++
11	8	Whole ocular region	MD	VI	0–20	++++	++++	+	+
12	8	Eyelid	MD	VI	0–20	++++	−	+	−
13	4	Third eyelid	MD	VI	0–20	++++	+++	+	−
14	9	Eyelid	MD	VI	0–20	++++	+	+	−
15	6	Third eyelid	PD	VI	30–60	++++	−	+	++++
16	7	Whole ocular region	PD	VI	30–60	++++	+	+	+
17		Eyelid	PD	VI	0–20	++++	+	−	++
18	8	Eyelid	PD	VI	>60	++++	+	+	+
19	10	Third eyelid	PD	VI	0–20	++++	++	+	−

* Multiple samples were evaluated from each case; ** WD = Well differentiated; MD = Moderately differentiated; PD = Poorly differentiated; ^‡^ MI = Moderately Invasive; NI = Non-Invasive; VI = Very Invasive; ^§^ Number of mitotic figures per ten high-power field, Low = 0–20; Medium = 30–60; High = >60; ^†^ − = absence of labelling; + = <25% of cells labelled; ++ = 25–50% of cells labelled; +++ = 50–75% of cells labelled; ++++ = >75% of cells labelled.
